# Drone versus ambulance for blood products transportation: an economic evaluation study

**DOI:** 10.1186/s12913-021-07321-3

**Published:** 2021-12-05

**Authors:** M. A. Zailani, R. Z. Azma, I. Aniza, A. R. Rahana, M. S. Ismail, I. S. Shahnaz, K. S. Chan, M. Jamaludin, Z. A. Mahdy

**Affiliations:** 1 Faculty of Medicine, Universiti Kebangsaan Malaysia (UKM), Kuala Lumpur, Malaysia; 2 Queen Elizabeth II Hospital (QEHII), Ministry of Health (MOH), Kota Kinabalu, Malaysia; 3 Sabah Women and Children Hospital (SWACH), Ministry of Health (MOH), Kota Kinabalu, Malaysia

**Keywords:** Drone, Blood, Transportation, Malaysia, Economy

## Abstract

**Background:**

Medical transportation is an essential step in health care services, and includes ground, air and water transportation. Among the important uses of medical transportation is the delivery of blood products in the event of a clinical emergency. Drone technology is the latest technological advancement that may revolutionize medical transportation globally. Nonetheless, its economic evaluation is scant and insufficient, whilst its cost-effectiveness remains controversial. The aim of this study was to compare the cost-effectiveness of drone transportation versus the ambulance.

**Methods:**

The setting of the study was within a developing nation. An economic evaluation study of drone versus ambulance for emergency blood products transportation between the Sabah Women and Children Hospital (SWACH) and the Queen Elizabeth II Hospital (QEH2) on Borneo Island was conducted using the Cost-Effectiveness Analysis (CEA) technique. The total cost of each mode of transportation was calculated using the Activity Based Costing (ABC) method. Travel time was used as a denominator to estimate the Incremental Cost Effectiveness Ratio (ICER).

**Results:**

For one clinical emergency in SWACH, a round trip of blood products transportation from SWACH to QEH2 costs RM1,266.02 (USD307.09) when using the ambulance, while the drone costs RM1,313.28 (USD319.36). The travel time for the drone was much shorter (18 min) compared to the ambulance (34 min). The Cost-Effectiveness Ratio (CER) of ambulance transportation was RM37.23 (USD9.05) per minute whilst the CER of drone transportation was RM72.96 (USD17.74) per minute. The ICER of drone versus ambulance was − 2.95, implying an increase of RM2.95 in cost for every minute saved using a drone instead of an ambulance.

**Conclusion:**

Although drone transportation of blood products costs more per minute compared to the ambulance, the significantly shorter transport time of the drone offset its cost. Thus, we believe there is good economic potential for drone usage for blood products transportation in developing nations particularly if the drone price decreases and its operational lifespan increases. Our limitation of a non-clinical denominator used in this study leads to the recommendation for use of clinical outcomes in future studies.

**Supplementary Information:**

The online version contains supplementary material available at 10.1186/s12913-021-07321-3.

## Background

Medical transportation is a basic but essential step in a health care service system. It involves the movement of patients, medical personnel, equipment, and medical supplies such as blood products, medication and vaccines. Conventionally, medical transportation includes ground transportation such as ambulances and motorcycles, and air transportation such as helicopters [1]. Less commonly, water transportation such as catamarans and boats are also used for the delivery of medical necessities to remote islands [2]. Understanding the basics of medical transportation is important to enhance its efficiency and to enable effective communication between patients and health care providers, ensuring accessibility to health services.

Among the important utilities of a medical transportation system is the delivery of blood products such as packed red blood cells and platelets in times of emergency. These products are life-saving biological components used as an integral part of resuscitation in cases of massive bleeding and haemorrhagic shock be it traumatic injuries or obstetric hemorrhage [3]. Patients who suffer serious internal or external bleeding would deteriorate rapidly within minutes, with a significant risk of death. The most effective way of treating such profound blood loss and saving life is by transfusing the patient with blood. Hence, every minute spent on medical transportation is crucial in determining the patient’s outcome.

In the Fourth Industrial Revolution (4IR), the drone features prominently as a technological advancement that may enhance medical transportation [4]. The drone or unmanned aerial vehicle (UAV) have become increasingly popular in recent years with widespread applications including in medicine [5], military [6], agriculture [7] and the construction industry [8]. The steady progress of the drone technology has accelerated its multitude of advantages such as being time-saving [9], having high accessibility over challenging geographical terrains [10], and reducing carbon footprint and greenhouse gas emission [11]. Studies around the world have been conducted to unlock the true potential of this technology in improving health care services, for example the maintenance of blood samples integrity when flown by drones [12] and the ability to reduce the response time in incidents of out-of-hospital cardiac arrest [13].

Apart from expertise, infrastructure and regulations [14, 15], one of the main barriers to surmount is the question of cost-effectiveness of drone usage. Thus far, there is only one study that attempted to answer the question of cost-effectiveness of drone for medical products transportation, the finding of which showed that motorcycles were more cost-effective than short-range (< 65 km) drones [16]. This result, however, may not be applicable in all types of geographical terrains or traffic. Recognizing this issue, we set out to compare the cost-effectiveness of drone transportation with a more common contender, the ambulance.

## Methodology

### Study setting

The study setting is the state of Sabah on Borneo Island with a challenging terrain and traffic flow, as well as the highest rate of obstetric haemorrhage in Malaysia. Sabah is Malaysia’s second largest state covering a land area of 73,904 km^2^. It is located in the northern part of Borneo and is subdivided into four divisions. Sabah’s geographical features include vast tropical rainforests, sandy beaches, and hundreds of mountains. The population in Sabah consists of an estimated 3.9 million people, making it the third most populous state in Malaysia. Only a quarter of the Sabahan population live in the capital city, Kota Kinabalu, while three quarters live in rural areas including along the coastline and mountainous terrain [17].

**Fig. 1:**
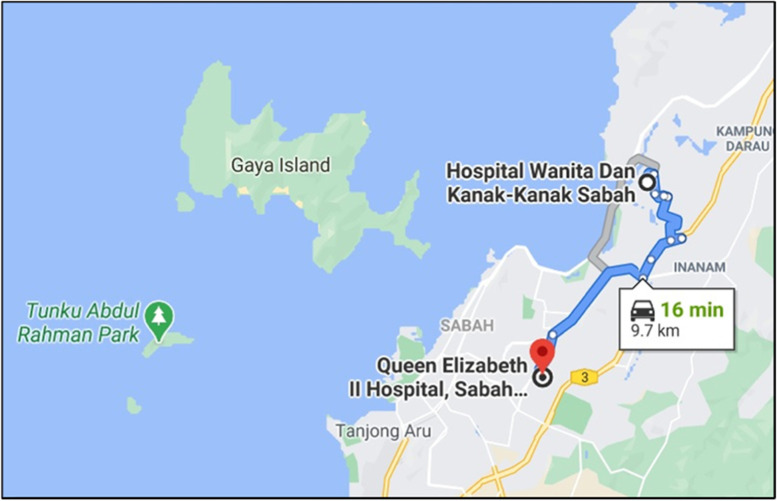
Google Maps of the location and distance between the Sabah Women and Children Hospital (SWACH) and Queen Elizabeth II Hospital (QEH2). (Source: Google, n.d. Retrieved on February 9, 2021, from https://goo.gl/maps/5BFABVv3V2sdgNC27)

The unique geography of Sabah and its demography lead to difficulties of access to health facilities, particularly for its rural population. The Sabah Women and Children Hospital (SWACH) is an example of a district hospital. It is located approximately 10 km from a tertiary hospital, Queen Elizabeth II Hospital (QEH2) (Fig. 1), which plays an important role for the SWACH’s case referrals and blood products supply. According to the 5th Report of the National Obstetrics Registry (NOR) Malaysia, SWACH was recorded as the hospital with the highest number of births in Malaysia (14,463 in 2017) [18] and is recognized as a Paediatric Thalassemia Center in Sabah. Ambulances are used to transport blood products from QEH2 to SWACH twice daily as there is limited blood storage in SWACH yet a high demand. For emergency cases, the average usage of SWACH’s ambulance for delivery of blood products is four times a month.

These multifactorial conditions in Sabah’s geography, with an example of usage of ambulances between SWACH and QEH2, highlighted a critical requirement of an alternative mode of blood products transportation in Sabah such as the drone.

### Study design

We conducted an economic evaluation study of the costs and cost-effectiveness of drone versus ambulance for the transportation of blood products. This analysis was done under a simulated public health emergency condition between the SWACH and the QEH2, a tertiary hospital that supplies the SWACH with blood when needed. Our denominator unit of effectiveness in the Cost-Effectiveness Analysis (CEA) of this study was the travel time during blood transportation.

We started the analysis with calculation of the total annual expenditure for the transportation of blood products using ambulance and drone respectively. The cost calculation was carried out using an Activity Based Costing (ABC) method (Tables 1 and 2). According to Aniza I. et al. (2019), the usage of this method will provide a rich source of information on the costing calculation, enable investigators to identify the individual component of the cost, and is suitable for a comparative intervention study in clinical and health care system management [19].

**Table 1:** Cost Calculation Model for Ambulance Transportation of Blood Products

Total cost for ambulance transportation = Capital cost + Recurrent cost Where: Capital cost = Vehicle cost Recurrent cost = Utility cost + Maintenance cost + Human resource cost (Medical Officer, Medical Laboratory Technologist, Health care Assistant and Driver) + Equipment cost + Disposable cost

**Table 2:** Cost Calculation Model for Drone Transportation of Blood Products

Total cost for drone transportation procedure = Capital cost + Recurrent cost Where: Capital cost = Vehicle cost Recurrent cost = Utility cost + Maintenance cost + Human resource cost (Medical Officer, Medical Laboratory Technologist, Drone Pilot, Drone Co-pilot) + Equipment cost + Disposable cost	

**Fig. 2:**
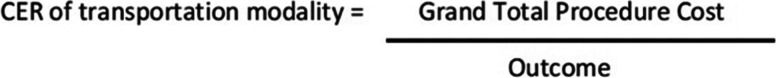
Equation for the calculation of Cost Effectiveness Ratio (CER)

Subsequently, the calculated total cost was used to determine a Cost-Effectiveness Ratio (CER), a ratio in which the net cost of an intervention was divided by the net changes in their health outcomes or effectiveness (Fig. 2) [20]. Joseph CG et al. (2000) in the Handbook of Statistics described CER as a useful economic tool for statistical evaluation of health care intervention, particularly in decision-making for policy-makers and stakeholders when faced with challenges in the allocation of health care funds across several competing interventions [21].

**Fig. 3:**
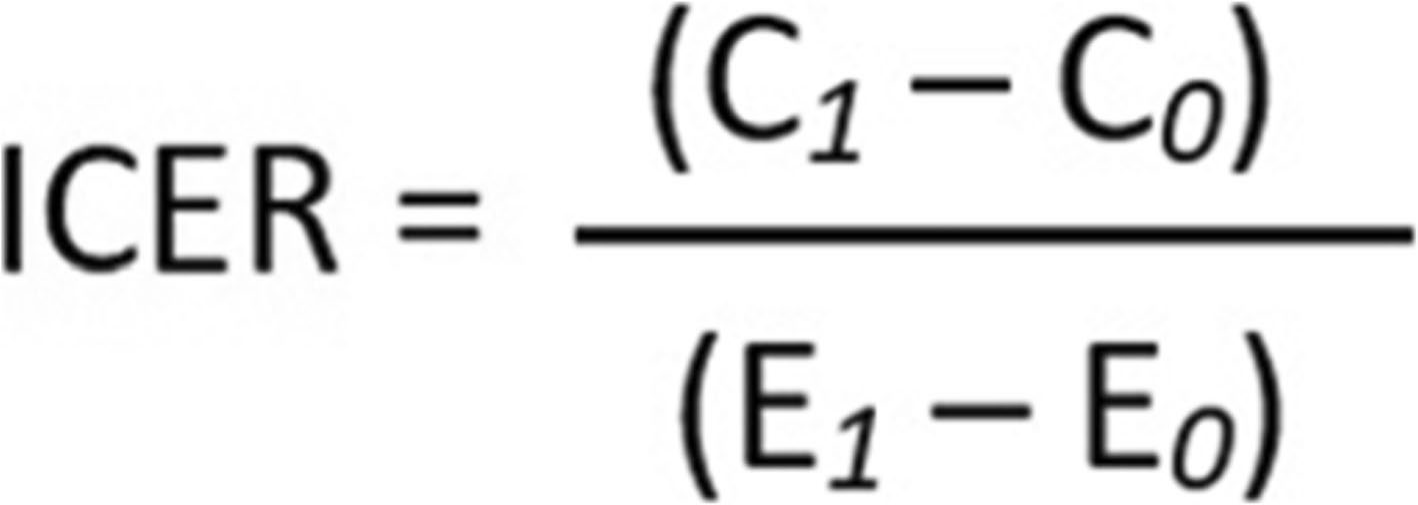
Equation for the calculation of Incremental Cost-Effectiveness Ratio (ICER)

Lastly, we summarized our economic findings by the analysis of the Incremental Cost-Effectiveness Ratio (ICER) and a sensitivity analysis. The ICER is defined as the difference in cost between two possible health interventions, divided by the difference in their effect or outcomes (Fig. 3).

For our study, ICER represents a summary of the economic value of medical drone intervention for blood products transportation, compared with its alternative (comparator) i.e. the conventional ambulance. The value of ICER is customarily the main output or result of any economic evaluation [22]. A sensitivity analysis was done to determine the robustness of our evaluation by examining the extent to which results are affected by changes in the dependent variable.

### Ethical approval

This study was registered under the National Medical Research Register (NMRR) of Malaysia and has obtained approval from the Medical Research and Ethics Committee (MREC), Ministry of Health, Malaysia (Reference Number: NMRR-19-1801-45,727 IIR) and the Universiti Kebangsaan Malaysia (UKM) Research Ethics Committee (Reference Number: UKM PPI.800-1/1/5/JEP.2019.420)**.**

### Data sources

We obtained ambulance operational data from the administration of the SWACH. These data include the frequency of blood products transportation between SWACH and QEH2 in a year, the average duration of a round trip, the flow chart of the procedure, the position and salary of the staff involved, the details of disposable items and equipment used for the procedure, the fuel consumption and the maintenance cost. All data were collected using an Activity Based Costing (ABC) form (Supplementary Material A).

For drone transportation, we obtained data of the costing from a professional drone operator (Aerodyne Group, Cyberjaya, Malaysia) [23]. The costing data represented a simulated drone flight between SWACH and QEH2 including the drone operational performance, salary details of human resources, putative prices, and maintenance cost of the drone. All drone data were collected using the ABC form (Supplementary Material B).

We obtained data for drone flight duration for a round trip between the SWACH and the QEH2 by conducting a simulated drone flight in Cyberjaya, Malaysia, the only established drone-flying zone in the country. In the simulated flight, we assumed a straight line (Euclidean distance between two points) of a drone flight from the SWACH to the QEH2 with an average distance of 8 km, flight altitude of 91 m above ground, flight velocity of 43.5 km per hour and flight payload of 1.55 kg.

### Vehicle models

Our calculation used data pertaining to the Toyota Hiace ambulance model manufactured in year 2018, a converted vehicle model that is most commonly used as ambulance in emergency response units including in the SWACH. The costing of the vehicle was recorded as at the time of year acquired by the hospital and the value were discounted according to the Table of Annualization Factor (Year 5, 5%) to gain the economic cost.

The drone used was the DJI Matrice 600 Pro (M600 Pro) manufactured in year 2020. This model was chosen based on its loading capacity and flight performance as befit the drone flight plan in our study with the purpose of transporting blood products between the SWACH and the QEH2. Similarly, the economic cost of the drone was obtained through discounting using the Table of Annualization Factor (Year 5, 5%) as performed on its comparator (ambulance).

## Results

### Cost calculation

The cost calculation for our economic evaluation study was carried out using the Malaysian local currency, Malaysian Ringgit (MYR), which was subsequently converted to USD using an online currency converter (https://xe.com) (accessed on 9 May 2021).

### Ambulance

#### Total cost per trip

##### Capital cost

Capital cost was defined as a fixed, one-time expense incurred on the purchase of necessities used in rendering services. We identified only one capital cost that was required for this transportation, which was the vehicle cost. For the ambulance, the cost was RM489,000.00. The annualization factor for vehicle cost is 4.329 (using Year 5, 5%). Therefore, the economic cost was RM112,959.11 (USD27,469.42). The average frequency of ambulance usage for blood products transportation between the SWACH and the QEH2 was 720 trips for non-emergency and 48 trips for emergency cases respectively, in a year. Therefore, the calculated vehicle cost for the ambulance per trip was RM147.08 (USD35.76).

##### Recurrent cost

Recurrent costs were the costs of maintaining and operating a given program or procedure. For the ambulance transportation of blood products between the SWACH and the QEH2, several recurrent costs were identified and included in the calculation, including:

*Utility cost*

The utility cost of the ambulance included its diesel fuel consumption, which was costed at RM2.18 (USD0.53) per litre. We assumed an average estimated distance travelled in a round trip by the ambulance between the SWACH and the QEH2 as 26.5 km in non-emergency and 18.5 km in emergency cases respectively. The route taken during non-emergency was longer due to a routine necessary detour. The estimated amount of diesel used by the ambulance was 1 l for every 9.8 km journey. Therefore, the calculated diesel fuel consumption cost was RM5.89 (USD1.43) per trip for non-emergency and RM4.16 (USD1.01) per trip for emergency cases respectively. In conclusion, the average utility cost was RM5.02 (USD1.22) per round trip.

*Maintenance cost*

The estimated maintenance cost of the ambulance was RM20,280.90 (USD4931.91) per year. Therefore, the average maintenance cost per trip for the ambulance was RM26.40 (USD6.41).

*Human resource cost*

The cost of human resource was calculated based on salary per minute for all personnel involved. This included two medical officers (MO), two medical laboratory technologists (MLT), one healthcare assistant (HA), and one ambulance driver. The calculated salaries per minute were RM0.43 (USD0.10) for the MO, RM0.04 (USD0.09) for the MLT, RM0.02 (USD0.005) for the HA, and RM0.02 (USD0.005) for the ambulance driver. The average travel time was 34 min. Therefore, the human resource cost was RM33.32 (USD8.10) per round trip, or RM0.98 (USD0.23) per minute.

*Equipment cost*

The cold-chain equipment used for ambulance transportation of blood products was a cool box for blood storage containing icepacks and a datalogger. The cost for the cool box (4 L Coleman box) was RM119.00 (USD28.93), RM24.00 (USD5.83) for the icepacks, and RM810.00 (USD196.97) for the datalogger (Fourtec MicroLite USB Datalogger LITE5032P-RH). Therefore, the total equipment cost was RM953.00 (USD231.75).

*Disposable cost*

The disposable cost was calculated by including all disposable items used routinely during the trip. The identified items were two pairs of Surgical Latex Rubber Gloves (Powdered) Size 5.5/ 6.0 at RM1.88 (USD0.45) per pair, and an average of 6 units of 450 ml blood bag at RM16.24 (USD3.94) per unit. Therefore, the total disposable cost was RM101.20 (USD24.60).

##### Grand total cost

The grand total cost per round trip for ambulance transportation was RM1,266.02 (USD307.87). The components of its calculation were summarized in Table 3.

**Table 3:** Calculation of the Total Cost for ambulance transportation of blood products between the Sabah Women and Children Hospital (SWACH) and the Queen Elizabeth II (QEH2) Hospital in Sabah, Malaysia

Costing Details	Average Cost per trip (MYR/USD)	Cost Division (%)
Capital cost		
Vehicle cost	147.08 / 35.76	11.62
Utility cost	5.02 / 1.22	0.40
Maintenance cost	26.40 / 6.41	2.08
Human resource cost	33.32 / 8.10	2.63
Equipment cost	953.00 / 231.75	75.28
Disposable cost	101.20 / 101.20	7.99
Grand Total Procedure Cost	1266.02 / 307.87	100.00

### Drone

#### Total cost per trip

##### Capital cost

The capital cost for blood products transportation using the drone was limited to vehicle cost only. The vehicle used was a DJI Matrice 600 Pro (M600 Pro) drone, which cost RM28,000.00 (USD6809.04). The annualization factor for vehicle cost was 4.329 (using Year 5, 5%). Therefore, the economic cost was RM6,468.00 (USD1572.89). We made the assumption that drone transportation was used for emergency cases only in view of its novelty. The average number of drone trips in a year was therefore 48 trips. Hence, the calculated vehicle cost for the drone was RM134.75 (USD32.76) per trip.

##### Recurrent cost

The recurrent cost of drone transportation included the following:

*Utility cost*

The calculation of utility cost was not applicable to the drone as there was no diesel fuel consumption. The power source of the drone came from six pieces of LiPo 6S lithium batteries (Model: TB47S) with a capacity of 4500 mAh, which was already included in the following calculation of the drone’s annual maintenance cost.

*Maintenance cost*

The maintenance cost of the drone included the lithium batteries as its power source. The estimated maintenance cost of the drone provided by the Aerodyne Group was RM5,000.16 (USD1215.94) per year [23]. Therefore, the calculated maintenance cost for the drone was RM104.17 (USD25.33) per trip.

*Human resource cost*

The cost of human resource was calculated using the calculated salary per minute of all personnel involved. This included two medical officers (MO), two medical laboratory technologists (MLT), one drone pilot and one co-pilot. The calculated salaries per minute were RM0.43 (USD0.10) for the MO, RM0.04 (USD0.01) for the MLT, RM0.10 (USD0.02) for the drone pilot, and RM0.08 (USD0.01) for the co-pilot. The average travel time for the drone was 18 min (with an average speed of 53.3 km per hour). Therefore, the human resource cost was RM20.16 (USD4.90) per trip, or RM1.12 (USD0.27) per minute.

*Equipment cost*

We envisioned that the cold chain equipment used for drone transportation was similar to the ambulance. The equipment was safely mountable on the drone and included a cool box for blood storage containing icepacks and a datalogger. Based on the calculation for the ambulance, the total equipment cost was RM953.00 (USD231.75).

*Disposable cost*

Assuming the cost was the same as for the ambulance, the total disposable cost was RM101.20 (USD24.60).

##### Grand total cost

The Grand Total Cost for drone transportation was RM1,313.28 (USD319.36). The components of its calculation were summarized in Table 4.

**Table 4:** Calculation of the Total cost for drone transportation of blood products between the Sabah Women and Children Hospital (SWACH) and the Queen Elizabeth II Hospital (QEH2)

Costing Details	Average Cost per trip (MYR/USD)	Cost Division (%)
Capital cost		
Recurrent cost	134.75 / 32.76	10.26
Utility cost	0 / 0	0
Maintenance cost	104.17 / 25.33	7.93
Human resource cost	20.16 / 4.90	1.54
Equipment cost	953.00 / 231.75	72.57
Disposable cost	101.20 / 24.60	7.70
Grand Total Procedure Cost	1313.28 / 319.36	100.00

For each clinical emergency in the SWACH, a round trip of blood products transportation from the SWACH to the QEH2 using an ambulance was estimated to cost RM1,266.02 (USD307.87). This was lower compared to the drone, which stood at RM1,313.28(USD319.36). Most of the cost for both transportation modalities was contributed by the equipment cost (75.28% for the ambulance and 72.57% for the drone). However, the vehicle cost for drone transportation contributed less to the Grand Total Cost compared to ambulance transportation (10.26% for drone versus 11.62% for ambulance).

### Outcome

The outcome parameter in our study was limited to travel time. Ambulance transportation of blood products between the SWACH and the QEH2 took 34 min per round trip, whilst the drone impressively took only 18 min per round trip, i.e. the drone required only a little more than half (52.94%) of the length of time taken by the ambulance.

### Cost-effectiveness ratio (CER)

**Fig. 4:**
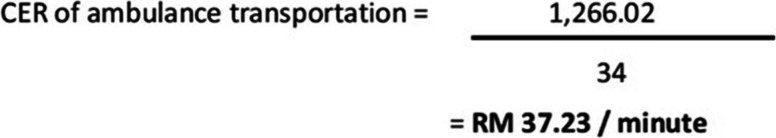
Calculation of the Cost-Effectiveness Ratio (CER) of ambulance transportation of blood products between the Sabah’s Women and Children’s Hospital (SWACH) and the Queen Elizabeth II Hospital (QEH2)

**Fig. 5:**
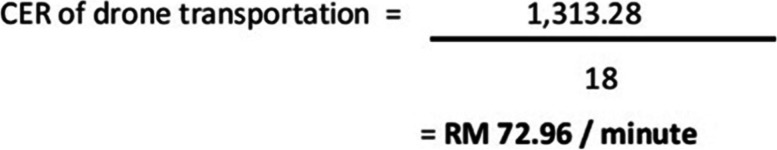
Calculation of the Cost-Effectiveness Ratio (CER) of drone transportation of blood products between the Sabah Women and Children Hospital (SWACH) and the Queen Elizabeth II Hospital (QEH2)

The CER of both transportation modalities were calculated by dividing the Grand Total Procedure Cost by the outcome (travel time). The calculations of CER for both modes of transportation are shown in Figs. 4 and 5.

The result of the calculated CER showed that ambulance transportation of blood products between the SWACH and the QEH2 cost RM37.23 (USD9.05) per minute of travel. Meanwhile, drone transportation of blood products cost a higher amount of RM72.96 (USD17.74) per minute of travel.

### Incremental cost-effectiveness ratio (ICER)

**Fig. 6:**
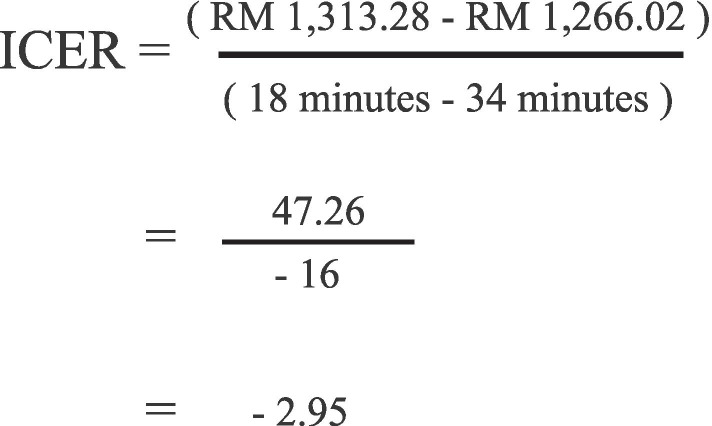
Calculation of the Incremental Cost-Effectiveness Ratio (ICER) of the drone versus the ambulance transportation of blood products between the Sabah Women and Children Hospital (SWACH) and the Queen Elizabeth II Hospital (QEH2)

The ICER was calculated using the equation in Fig. 2 where C_*1*_ and E_*1*_ are the Grand Total Procedure Cost and the effect of the intervention group (drone transportation of blood products) respectively, and C_*0*_ and E_*0*_ are the Grand Total Procedure Cost and the effect of the comparator group (ambulance transportation of blood products) respectively. Our results demonstrated an ICER value of – 2.95 (Fig. 6).

From our calculation, it can be inferred that by using the drone for transportation of blood products, an additional cost of RM47.26 (USD11.30) is needed to reduce 16 min of travel time, which equals an increment of RM2.95 (USD0.70) to reduce 1 min of travel time between the SWACH and the QEH2. Our calculation revealed that the drone cost more, but this was compensated by the shorter travel time, which may be life-saving in an emergency.

### Sensitivity analysis

A sensitivity analysis is an analysis of several case scenarios [24] to apportion changes in the output of a proposed system or solution, which for this study is the drone transportation. The scenarios were base-, best- and worst-case scenarios. Calculation of total cost for all scenarios was based on the total number of trips.

The base-case scenario for our study was calculated by multiplying the number of trips by the cost, which was 48 x RM1,313.28 (USD319.36), yielding RM63,037.44 (USD15,329.28). The best-case scenario (increasing drone trips by 50%) produced RM94,556.16 (USD22,993.92), whereas the worst-case scenario (reducing drone trips by 50%) showed the cost to be RM31,518.72 (USD7,664.64).

## Discussion

Globally, we found exceptionally few scientific studies that reported on the economic perspective of drone technology in healthcare services [13, 16]. This may be due to the slow-paced implementation of drone usage for medical purposes. Thus, our pilot study is the first in Malaysia to evaluate the drone’s economic viability. We strived to provide evidence-based answers with regard to the economic feasibility of using the drone as a potential vehicle for emergency blood products transportation between district and tertiary hospitals. The findings of our study in Sabah can also be extrapolated to other parts of Malaysia, as well as other Southeast Asian countries that share similar economic status, climate, and topography.

Our economic evaluation of drone transportation of blood products revealed that the drone cost more than the ambulance for deployment in emergencies between a district hospital and the nearest tertiary hospital. This conclusion was made based on i) the higher CER calculation of the drone, which was RM72.96 (USD9.05) per minute of travel, compared to the CER of ambulance, which was RM37.23 (USD17.74) per minute of travel; and ii) a negative ICER value of – 2.23. Nonetheless, this is offset by the drone by nearly halving the travel time as a result of the straight route of travel, and absence of ground hindrances such as traffic congestion.

This impactful finding can be translated into a huge potential for drone technology to be used as a mode of blood products transportation in developing countries such as Malaysia in the future, particularly when the drone market matures and the drone price drops. Moreover, technological progress will optimize the drone’s operational lifespan, capacity, and capability, with consequent reduction in its maintenance cost. This promising potential is reflected in the outcome achieved in our economic evaluation of a simulated transportation of blood products between the SWACH and the QEH2, where the drone was able to reduce the travel time between the two hospitals.

Our proposal to use the drone in providing healthcare services is supported by a study conducted by Claesson et al. (2016) in which their simulation model showed that drones were capable of arriving at a faster rate before the conventional emergency response system (ambulance) in 93% of cases in rural areas [13]. However, the simulation did not analyse nor report the economic impact of the drone as it was not the aim of their study. Owing to the poor geographical terrain of rural Sabah, the shorter travel time required by the drone compared to ground vehicles is advantageous. A similar argument may be applicable in urban areas with high traffic flow such as Kuala Lumpur, the capital city of Malaysia. In health facilities with limited blood storage, efficient emergency transportation by drone obviates the need for considerable peripheral blood storage for emergency purposes, hence eliminating wastage of blood supply as a result of product expiry. An excellent proven example of such benefit is found in the Rwandan Zipline system [25].

### Limitation

Our economic evaluations were based on drone simulation flights in a drone fly zone outside Sabah, as it was legally untenable to conduct the flight in Sabah itself. Sabah local authority bylaws currently impose strict prohibition against drone flight, citing concerns with regard to safety of civilians. Specific authorisation that is hard to obtain, is required in order to fly the drone between the two hospitals, thus severely compromising our chances of accomplishing such a feat.

We overcame the limitation with simulated drone flights in an airspace in Cyberjaya where drone flights were freely permissible, with a similar Euclidean distance in order to observe the duration and outcome of the process. In future, we plan to conduct a prospective study using drone transportation in the real geographical location in Sabah in order to support our calculation.

Another limitation encountered by our study was the absence of a clinical outcome as a denominator in the calculation of ICER. As an example, the number of transportations of blood products aided by drone would be a more suitable clinical denominator for the ICER calculation. We plan to adopt such clinical parameters as our outcome measure in future studies following this pilot project. Currently there is very little clinical outcome data available globally for studies on drone transportation. From our own experience, it is difficult to obtain ethical approval for such studies at the moment, given the current challenges surrounding flight authority approval, doubtful public acceptance, and the strict requirement for licensing of drone flights beyond visual line of sight (BVLOS).

## Conclusion

Our economic evaluation concluded that, although drone transportation of blood products cost more as compared to ambulance, the significantly reduced travel time as an outcome measure offset the cost. Therefore, from an economic viewpoint, the drone is a more cost-effective and viable mode of blood products transportation particularly during emergencies.

The findings of this study add to the body of knowledge pertaining to the cost-effectiveness of the drone as a vehicle for healthcare service delivery. We focused on one of the potential usages of the medical drone where time is of the essence, namely blood and blood products transportation.

## Supplementary Information

**Additional file 1 **: **Supplementary material A**. Activity Based Costing (ABC) form for ambulance. **Supplementary material B**. Activity Based Costing (ABC) form for drone.

## Data Availability

All data generated or analysed during this study are included in this published article (and its supplementary information files).

## Abbreviations

4IR: Fourth Industrial Revolution
UAV: Unmanned Aerial Vehicle
SWACH: Sabah’s Women and Children Hospital
QEH2: Queen Elizabeth II Hospital
UKM: Universiti Kebangsaan Malaysia
CEA: Cost-Effectiveness Analysis
ABC: Activity Base Costing
CER: Cost-Effectiveness Ratio
ICER: Incremental Cost-Effectiveness Ratio
MYR: Malaysian Ringgit
USD: United States Dollar
