# Supplementary material for: Drone versus ambulance for blood products transportation: an economic evaluation study
Source: BMC Health Serv Res. 2021 Dec 5;21:1308. doi: 10.1186/s12913-021-07321-3 (PMC8645114; doi:10.1186/s12913-021-07321-3)

**SUPPLEMENTARY MATERIAL A**

Activity Based Costing (ABC) form for ambulance.


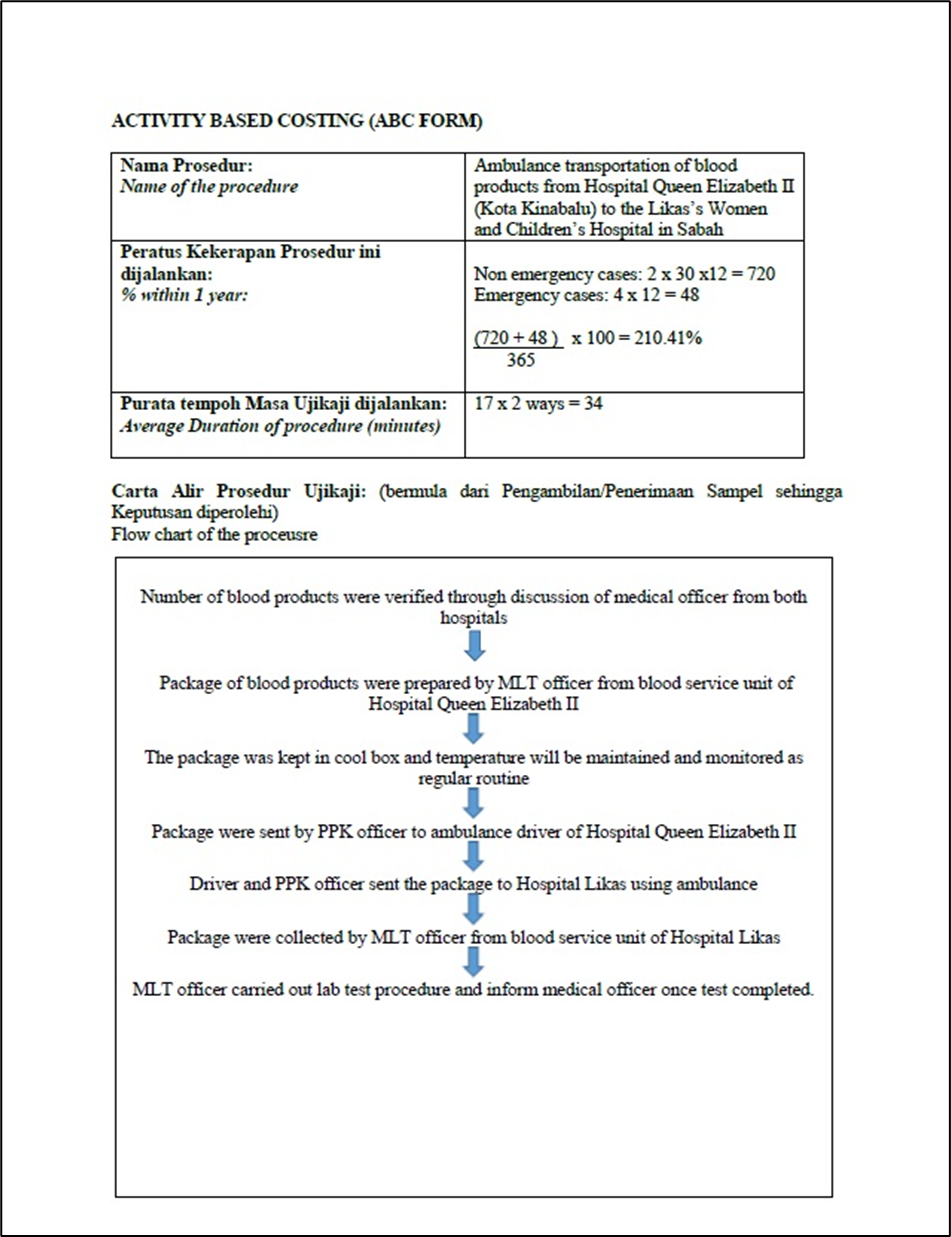


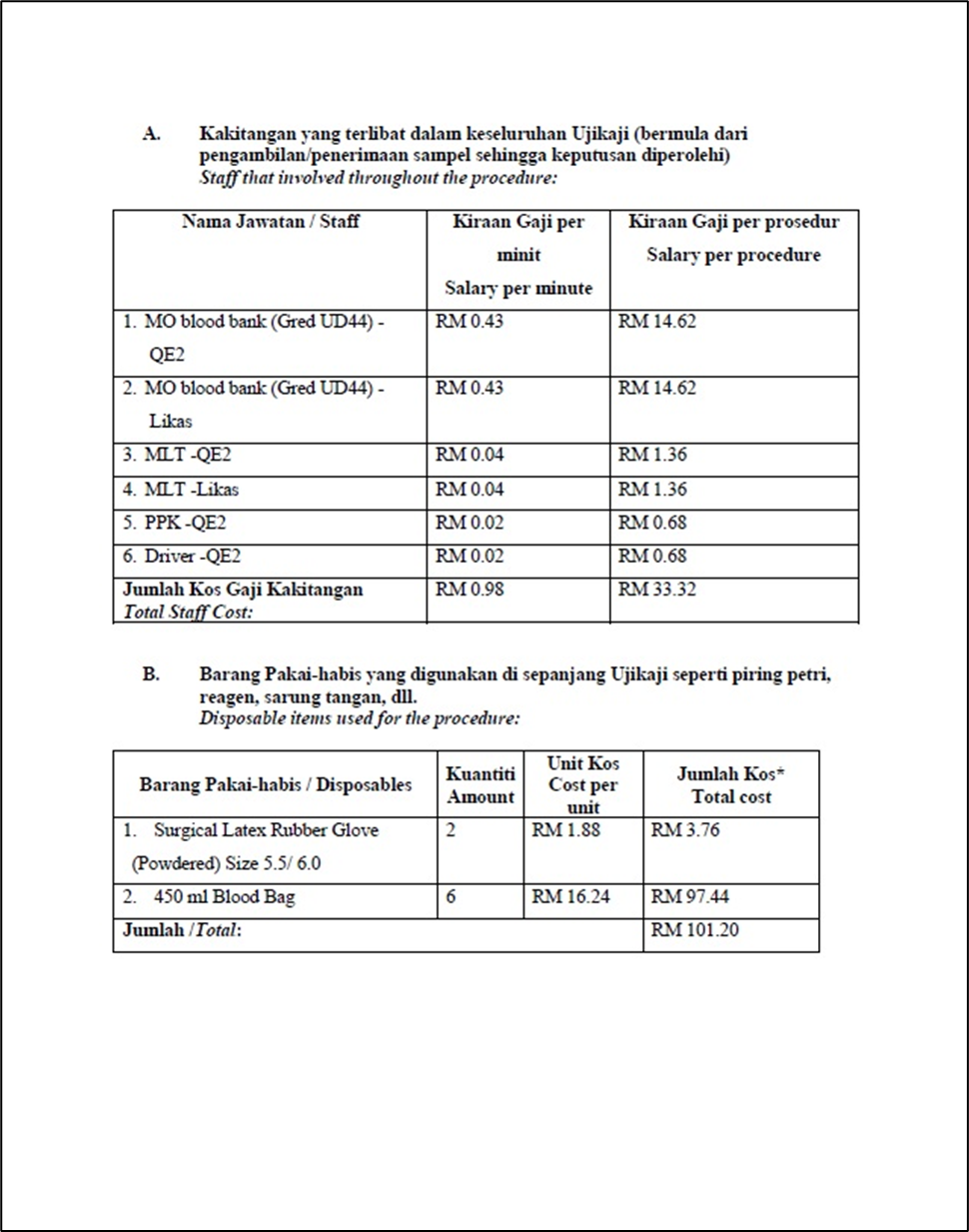


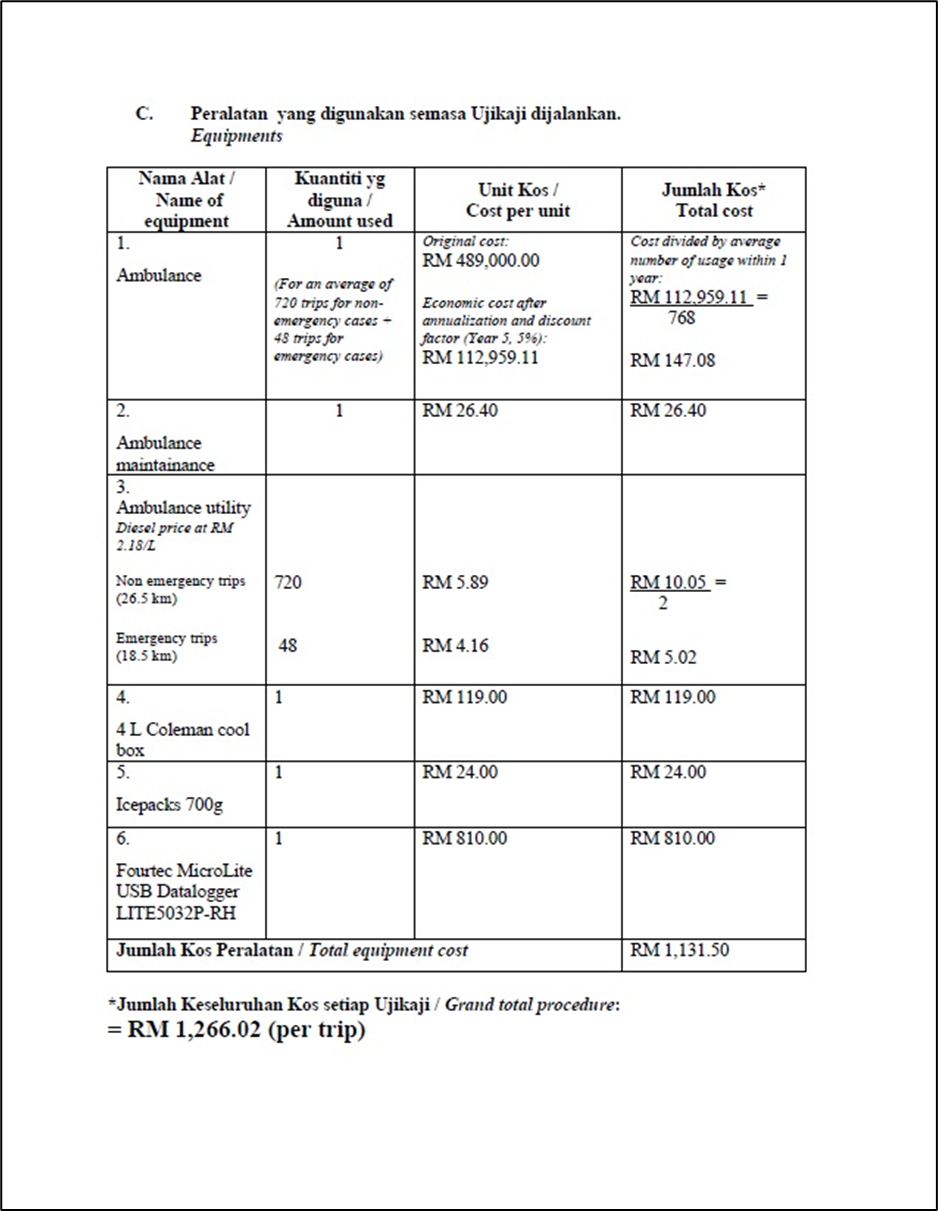


**SUPPLEMENTARY MATERIAL B**

Activity Based Costing (ABC) form for drone.


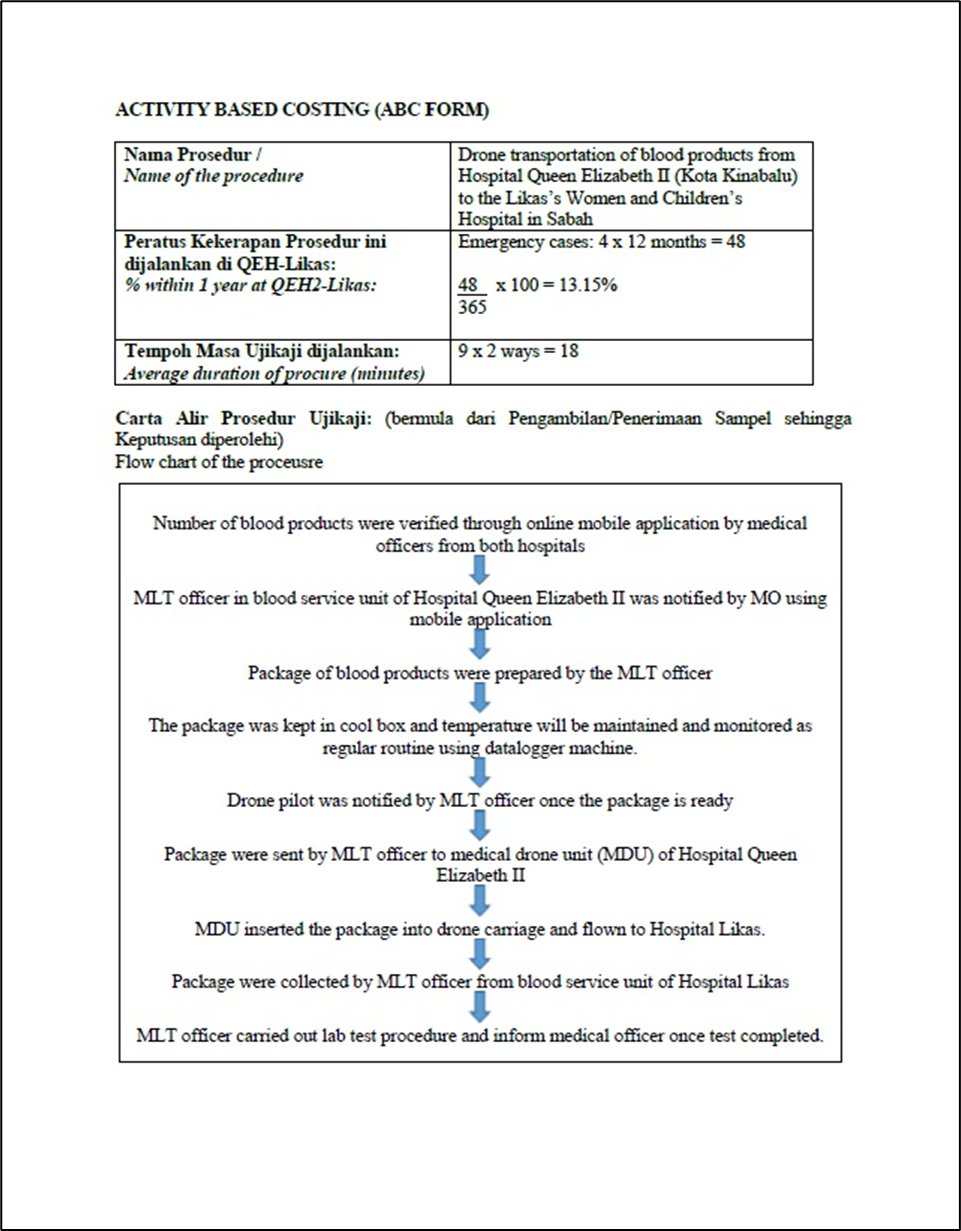


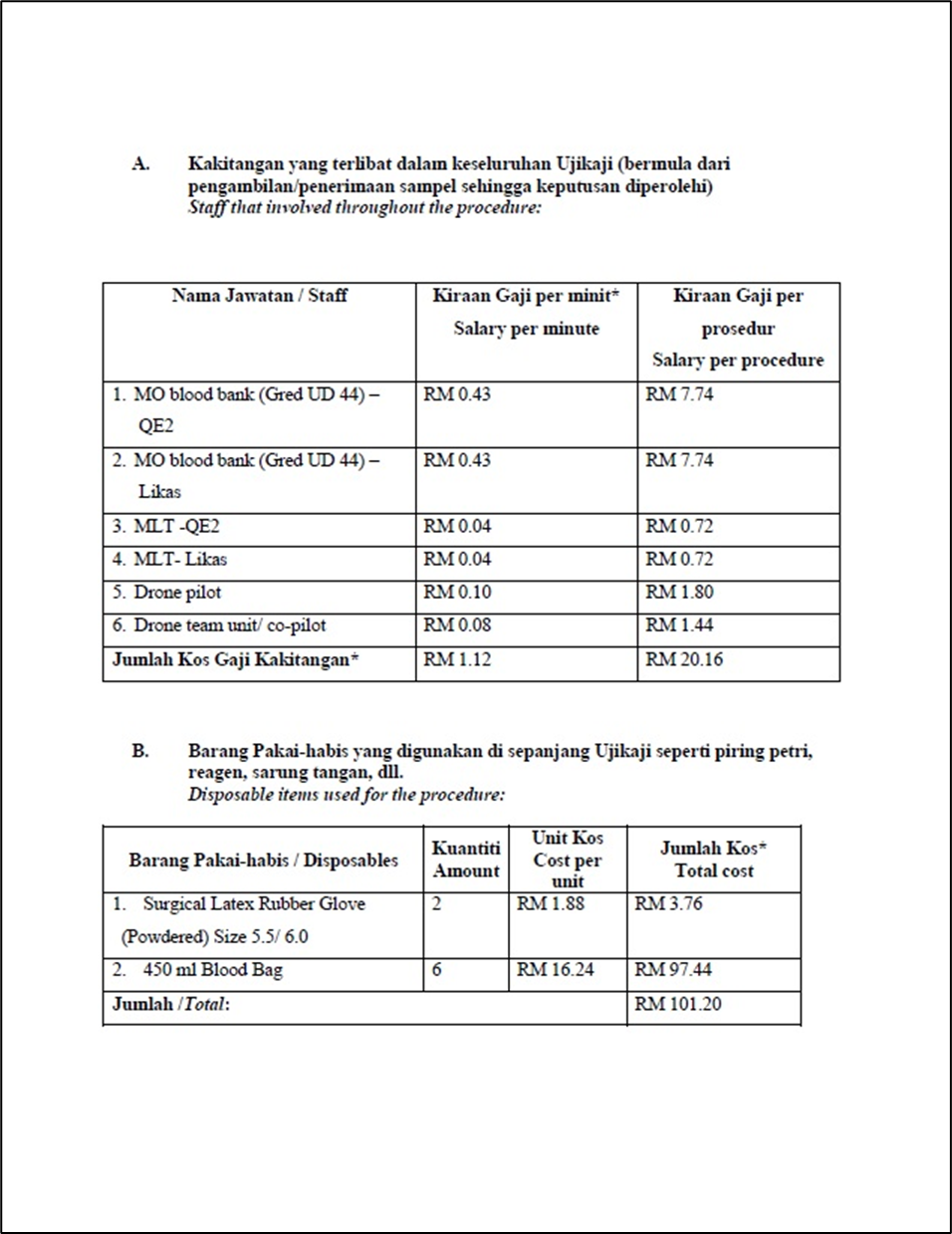


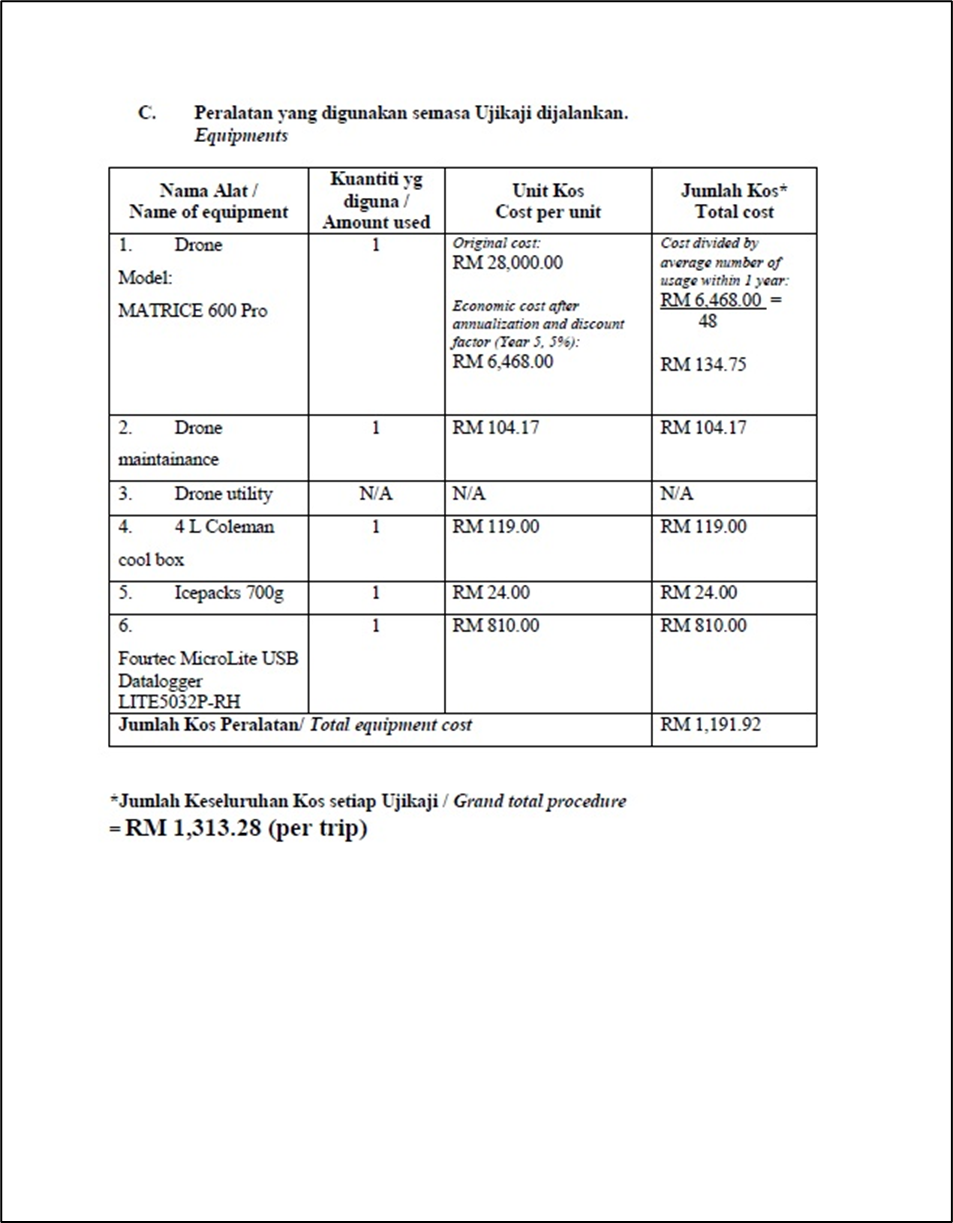

Supplement: Supplementary file 1 — Additional file 1 : Supplementary material A. Activity Based Costing (ABC) form for ambulance. Supplementary material B. Activity Based Costing (ABC) form for drone. [file 12913_2021_7321_MOESM1_ESM.docx]
